# Gene Expression Profiles at Moxibustioned Site (ST36): A Microarray Analysis

**DOI:** 10.1155/2013/890579

**Published:** 2013-09-22

**Authors:** Hai-Yan Yin, Yong Tang, Sheng-Feng Lu, Ling Luo, Jia-Ping Wang, Xu-Guang Liu, Shu-Guang Yu

**Affiliations:** ^1^Acupuncture & Tuina School, Chengdu University of Traditional Chinese Medicine, Chengdu 610075, China; ^2^Joint Laboratory of Biochip between Chengdu University of Traditional Chinese Medicine and CapitalBio Co. Ltd., 37 Shi-er Qiao Road, Chengdu 610075, China; ^3^Key Laboratory for Acupuncture & Chronobiology of Sichuan Province, Chengdu 610075, China; ^4^Acupuncture & Tuina School, Nanjing University of Traditional Chinese Medicine, Nanjing 210029, China; ^5^Laboratory for Acupuncture & Systematic Biology, Chengdu University of Traditional Chinese Medicine, Chengdu 610075, China

## Abstract

As a major alternative therapy in Traditional Chinese Medicine, it has been demonstrated that moxibustion could generate a series of molecular events in blood, spleen, and brain, and so forth. However, what would happen at the moxibustioned site remained unclear. To answer this question, we performed a microarray analysis with skin tissue taken from the moxibustioned site also Zusanli acupoint (ST36) where 15-minute moxibustion stimulation was administrated. The results exhibited 145 upregulated and 72 downregulated genes which responded immediately under physiological conditions, and 255 upregulated and 243 downregulated genes under pathological conditions. Interestingly, most of the pathways and biological processes of the differentially expressed genes (DEGs) under pathological conditions get involved in immunity, while those under physiological conditions are involved in metabolism.

## 1. Introduction

In acupuncture research, microarray analysis has been widely employed to uncover gene expression profiles at different tissues or organs [1–19]. Based on these gene expression profiles, researchers would be able to have the possibility to find out more potentially interesting targeted genes to conduct further experiment to explain the molecular events induced by acupuncture. Moxibustion, as one of the main therapies in acupuncture clinical practice, has been demonstrated to it could be useful for pain relief [20, 21] and generated a series of molecular events in blood [22, 23], spleen [24, 25], colonic mucosa [26], brain [27], and so forth, by utilizing moxa cone or stick to stimulate acupoint or some areas (also named moxibustioned site). However, none of gene expression profiles at moxibustioned site to date has been reported. Therefore, we proposed that moxibustion could, to a considerable extent, yield a great deal of differentially expressed genes (DEGs) at moxibustioned site, and we also anticipate to find out potential molecular targets to explain how moxibustion works at the stimulated site.

## 2. Material and Methods

### 2.1. Animals

Adult male Sprague-Dawley rats weighing 200–220 g obtained from Chengdu University of Traditional Chinese Medicine, Experimental Animal Centre, were utilized in this study. Maintained in animal room of automatically controlled day cycles (12 : 12 = light : dark cycle) at 24 ± 2°C, all rats were allowed to freely take food and water *ad libitum *and randomly assigned to the various experimental groups (*n* = 3, for each group). The experimental procedures were conducted in accordance with the National Institutes of Health (NIH) Guide for the Care and Use of Laboratory Animals, and all experimental protocols were approved by the Animal Ethics Committee of Chengdu University of Traditional Chinese Medicine.

### 2.2. Experimental Design

In this study, we aimed to explore what would take place at the moxibustioned site in the view of potential molecular target under physiological and pathological conditions. Thus, we designed two different parts of microarray experiment. One is designed for uncovering the gene expression profile at physiological condition in which it consisted of 2 group: healthy control group (C) and healthy control with moxibustion stimulation group (CM). The other one is designed for revealing the gene expression profile at pathological condition in which it composed of model control group (M) and model control with moxibustion stimulation group (MM).

### 2.3. Intervention

#### 2.3.1. Physiological Condition

**Figure 1:**
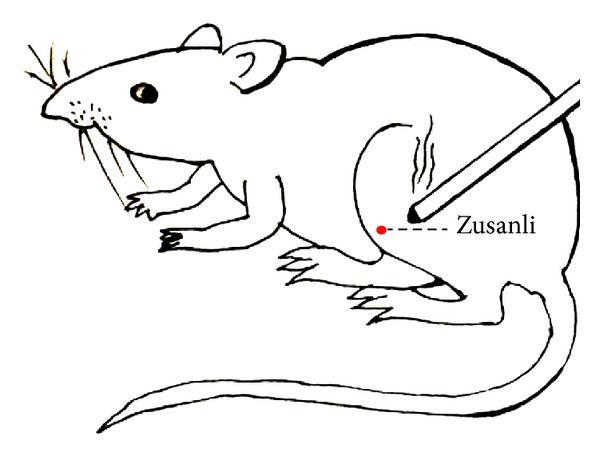
Rat received moxibustion at ST36 (Zusanli).

Under physiological condition, the rats in CM group received moxibustion at the left acupoint Zusanli (ST36), at the depression below the knee from the anterior crest of the tibia [28] for 15 min. The moxibustion stimulation was manipulated with lighting moxa stick (length: 12 cm, diameter: 0.6 cm, Nanyang Hanyi Moxibustion Technology Development Co., Ltd., China) for 15 min (Figure 1). In case of skin burnt, the tip of moxa stick was kept about 2-3 cm from the skin.

#### 2.3.2. Pathological Condition

Firstly, the pathological condition was established by injecting subcutaneously with 0.1 mL Freund's Complete Adjuvant (FCA, Sigma, USA) into the plantar surface of the left hind paw of the rat [29]. The CFA injection immediately led to local inflammation, paw swelling and pain, which became apparent within 12 hours and persisted for at least 2 weeks after injection. In this experiment, the rats in MM group received moxibustion with the same procedure as mentioned above 1 week after injection.

### 2.4. RNA Extraction

Two hours after one time of moxibustion stimulation was completed, rats was euthanized by CO_2_ inhalation. The cutaneous tissue (0.5 cm × 0.5 cm × 0.2 cm) located at moxibustioned site were immediately removed and preserved in RNAlater (Ambion, USA) to prevent RNA degradation. Total RNA was extracted using TRIzol Reagent (Invitrogen, USA) and purified with RNA clean-up Kit (MN, Germany) following the instructions of manufacturers, respectively. Total RNA was quantitated by spectrophotometry, and the integrity was assessed by formaldehyde denatured agarose gel electrophoresis. 

### 2.5. Microarray Analysis

The microarray analysis service provided by CapitalBio Corporation (Beijing, China) was performed as described [30, 31]. Briefly, total RNA extracted from the samples was used to produce complementary RNA using in vitro transcription technique. Then cDNA was generated by reverse transcription and used as the template to synthesize the fluorescein-labeled cDNA by Klenow fragment polymerase. Universal rat reference RNA purchased from Stratagene was also labeled as common reference control. RNA from sample and common reference were fluorescently labeled by Cy5 or Cy3, respectively, and then were hybridized paired to 27K Rat Genome Array (CapitalBio, China). The array was comprised of 26,962 oligonucleotide probes covering 27,044 transcripts which represent about 22,012 genes. All arrays were scanned by LuxScan 10KA dual channel confocal laser scanner (CapitalBio, China). The obtained images were analyzed with LuxScan3.0 Image Analysis Software (CapitalBio, China), which employed the LOWESS normalization algorithm.

### 2.6. Data Analysis

#### 2.6.1. Differentially Expressed Genes Selection

The detected signal intensities of all probes on the chip ≥400 were included for comparison analysis. We applied two-class unpaired algorithm in the Significant Analysis of Microarray software (SAM, Stanford) to identify significantly differentially expressed genes between CM and C groups, and MM and M groups. DEGs were determined with the threshold of false discovery rate, FDR ≤5% and fold change ≥2.0 or ≤0.5.

#### 2.6.2. Pathway and Biological Processes Analysis of DEGs

We employed the online Molecule Annotation System (MAS) established by CapitalBio Corporation (http://bioinfo.capitalbio.com/mas3/) which integrated with KEGG and Gene Ontology (GO) database to perform pathway and GO Biological Process term enrichment analysis and calculate the statistical significance as described [32]. *P*  value <0.001 was considered statistically significant.

### 2.7. Real Time PCR Confirmation

To validate the expression patterns obtained from microarray data, we used quantitative real time polymerase chain reaction (qPCR) to detect the expression of four DEGs, Hspa1a, Mcpt8, Slpi, and Clqa, which were randomly selected from the 27K Rat Genome Array.  Table 1 showed the primers designed for these genes and the housekeeping gene Gapdh. cDNA was prepared from DNase-treated total RNA using the First Strand SuperScript II Kit (Invitrogen, USA). qPCR was performed with DNA Master SYBR Green I Kit (Roche, Germany) and LightCycler machine (Roche, Germany) following the manufacturer's protocols.

## 3. Results

### 3.1. DEGs at Moxibustioned Site

Different numbers of DEGs at moxibustioned site were obtained from different condition. Under physiological condition, we obtained 145 upregulated and 72 downregulated DEGs (see Supplementary Table 1 in Supplementary Material available online at http://dx.doi.org/10.1155/2013/890579). While under pathological condition, the results displayed 255 upregulated and 243 downregulated DEGs (Supplementary Table 2).

### 3.2. Enriched Pathways at Moxibustioned Site

**Figure 2:**
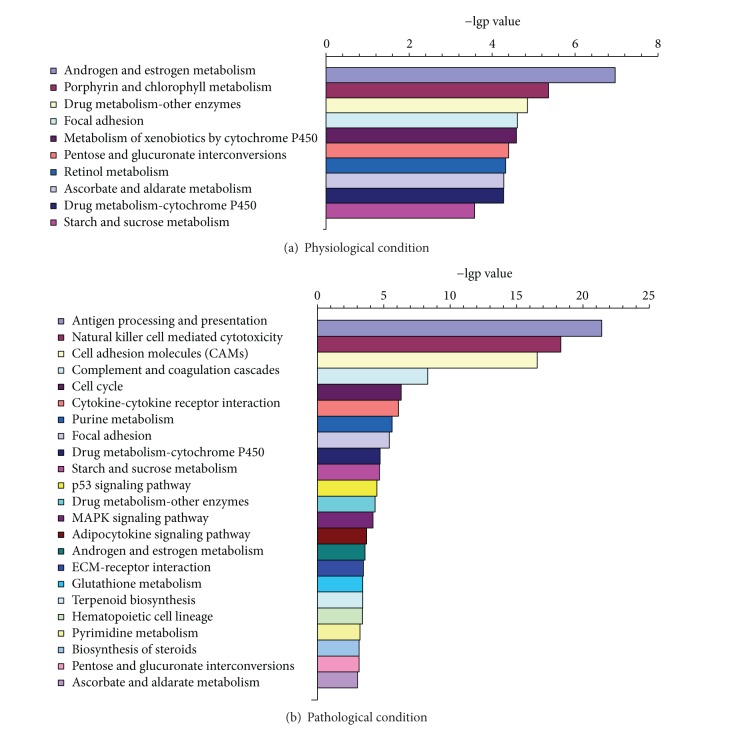
The statistically significant pathways (*P* value <0.001) involved in DEGs at moxibustioned site.

Figure 2 showed us statistically significant pathways (*P*  value <0.001) at moxibustioned site. Under physiological condition (Figure 2(a)), it was found that 10 pathways were enriched based on all DEGs at moxibustioned Site. On the other hand, 21 enriched pathways were statistically significant under pathological condition (Figure 2(b)).

### 3.3. Enriched Biological Processes at Moxibustioned Site

**Figure 3:**
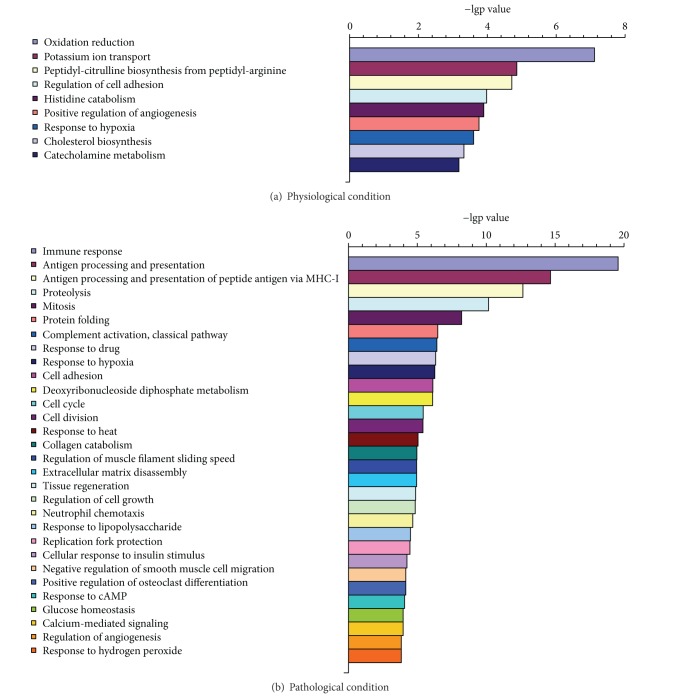
The statistically significant biological processes (*P* value <0.001) involved in DEGs at moxibustioned site.

From Figure 3, we would find out the biological processes with significantly statistical differences (*P*  value <0.001) at moxibustioned site. Under physiological condition (Figure 3(a)), it was found out that 9 biological processes were involved. Under pathological condition (Figure 3(b)), 29 biological processes were enriched.

### 3.4. Validation of the 4 Selected Genes

**Figure 4:**
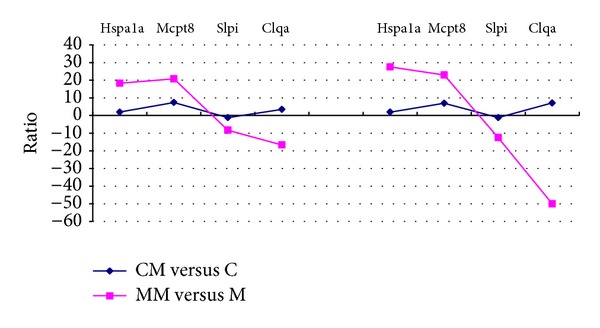
qPCR validation for 4 DEGs from microarray data.

To validate the results of the microarray, we selected 4 genes, Hspa1a, Mcpt8, Slpi, and Clqa, by qPCR. The results indicated that the expression levels of confirmed genes in microarray were similar with that in qPCR (Figure 4). 

## 4. Discussion

Here, we firstly reported the gene expression profiles at the moxibustioned site. The results imply that the moxibustioned site would also generate a setting of DEGs apart from those in other any tissue [16]. In other words, either under physiological condition or under pathological condition, we could find out a great number of DEGs responding to moxibustion stimulation at moxibustioned site. Based on these DEGs under different condition, we further seek out 22 coexpressed genes with similar expression tendency (Supplementary Table 3). To some extent, these genes (16 upnegulated and 6 downregulated genes) should be most likely to be considered as potential targets for continuing studies to determine which genes would be essential or critical in the role of moxibustion at moxibustioned site.

In this study, most (8/10) of the involved pathways at moxibustioned site were related to metabolism under physiological condition (Figure 2(a)). However, the significant pathways under pathological condition induced by FCA were most associated with immunity (Figure 2(b)), such as the pathway of antigen processing and presentation and natural killer cell-mediated cytotoxicity. It is suggested that different pathway at the moxibustioned site would get involved of the in different condition even with same moxibustion stimulation. 

According to the biological processes analysis, we also could find out the difference from different state. Without FCA as the pathological stimulation, the biological progresses following moxibustion administration at moxibustioned site were most composed of oxidation reduction, potassium ion transport, and so forth. However, a variety of biological processes related to immunity, such as immune response, antigen processing and presentation, and antigen processing and presentation of peptide antigen via MHC class I, and were exhibited in this study. 

Taken together, it seemed to be concluded that a series of molecular events would happen at moxibustioned site. Moreover, different pathways and biological progresses at moxibustioned site would be involved in different condition. However, this conclusion should be seriously taken for granted given the following limitations. Firstly, only one stimulation time point was used in this study. How about the time course or different time points, such as 5, 10, 15, 20, and 30 minutes, which were frequently practiced in moxibustion clinic, which needs to be answered in future study? Secondly, the pathological condition was induced by FCA injection in this study. To our knowledge, FCA injection will generate adjuvant arthritis through a series of immune actions [33, 34]. Moreover, previous studies also demonstrated that moxibustion would be useful to get better improved adjuvant arthritis [32, 35, 36]. Therefore, we cannot determine whether the DEGs at moxibustioned site under pathological condition would play important role in the moxibustion treatment of adjuvant arthritis. In this point of view, current data can only be used to explain what had happened at moxibustioned site under this condition. How about other pathological conditions? It is necessary to perform more researches to figure out the difference or similarity under different pathological conditions. 

Additionally, in this study the tissue used for RNA extraction and microarray detection was taken from the skin of the moxibustioned site. So far, the skin has been also regarded as an important immune organ [37, 38] as well as a component of neuro-immuno-cutaneous system (NICS) [39–42]. In view of this aspect, we would be able to assure that the molecular event at moxibustioned site in this experiment will have the possibility to be applied to explain the initial mechanism of moxibustion activating the neuroimmune modulation which has been demonstrated in previous studies [43, 44]. This would be another potential mechanism of moxibustion apart from that it is currently considered as temperature-related and non-temperature-related work mechanisms [45].

## 5. Conclusions

The results suggested that a set of molecular events would have happened at moxibustioned site. Among those molecular events, different genes and different pathways and biological progresses at moxibustioned site would have got involved under different conditions.

## Supplementary Material

Supplementary Table 1: The DEGs list under physiological condition (CM versus C)
Supplementary Table 2: The DEGs list under pathological condition (MM versus M)
Supplementary Table 3: Co-expressed genes list between physiological and pathological condition.

## Figures and Tables

**Table 1:** The primer designed for validation.

Gene	Primer (5′-3′)	Temperature (°C)	Product size (bp)
Gapdh	FW: CCTTGTAAGGGCAAAACCAA RV: ATGGCCTTCCGTGTTCCTAC	59	156
Hspa1a	FW: GGTGAACTACAAGGGCGAGA RV: GCTGCGAGTCGTTGAAGTAG	58	152
Mcpt8	FW: CCAGGTCATCGCTGTTGTAAA RV: CCCAGGTTTCACCCAGTCC	62	382
Slpi	FW: ACAGACAGGGGCTCTCTTGA RV: CCTCCCAATAAGTGCCAGAA	60	216
Clqa	FW: AAGTGGGACCTTTGTCTGTCTATC RV: CCCTGCTAACACCTGGAAGAG	59	108

## References

[1] Kim SK, Kim J, Ko E (2011). Gene expression profile of the hypothalamus in DNP-KLH immunized mice following electroacupuncture stimulation. *Evidence-Based Complementary and Alternative Medicine*.

[2] Choi Y-G, Yeo S, Hong Y-M, Lim S (2011). Neuroprotective changes of striatal degeneration-related gene expression by acupuncture in an MPTP mouse model of Parkinsonism: microarray analysis. *Cellular and Molecular Neurobiology*.

[3] Choi Y-G, Yeo S, Hong Y-M, Kim S-H, Lim S (2011). Changes of gene expression profiles in the cervical spinal cord by acupuncture in an MPTP-intoxicated mouse model: microarray analysis. *Gene*.

[4] Choi Y-G, Yeo S, Hong Y-M, Lim S (2011). Neuroprotective changes of striatal degeneration-related gene expression by acupuncture in an MPTP mouse model of Parkinsonism: microarray analysis. *Cellular and Molecular Neurobiology*.

[5] Tan C, Wang J, Feng W, Ding W, Wang M (2010). Preliminary correlation between warm needling treatment for knee osteoarthritis of deficiency-cold syndrome and metabolic functional genes and pathways. *Journal of Acupuncture and Meridian Studies*.

[6] Jiang L-H, Wang L-L (2010). Gene chips-aided analysis on the profiles of hippocampal whole-genome expression in depression rats following electroacupuncture treatment. *Zhen Ci Yan Jiu*.

[7] Hong MS, Park H-K, Yang J-S (2010). Gene expression profile of acupuncture treatment in 1-methyl-4-phenyl-1,2, 3,6- tetrahydropyridine-induced Parkinson’s disease model. *Neurological Research*.

[8] Sohn S-H, Kim SK, Ko E (2010). The genome-wide expression profile of electroacupuncture in DNP-KLH immunized mice. *Cellular and Molecular Neurobiology*.

[9] Kim SK, Park JY, Koo BH (2009). Adenoviral gene transfer of acetylcholinesterase T subunit in the hypothalamus potentiates electroacupuncture analgesia in rats. *Genes, Brain and Behavior*.

[10] Shiue H-S, Lee Y-S, Tsai C-N, Hsueh Y-M, Sheu J-R, Chang H-H (2008). DNA microarray analysis of the effect on inflammation in patients treated with acupuncture for allergic rhinitis. *Journal of Alternative and Complementary Medicine*.

[11] Wang X-Y, Li X-L, Hong S-Q, Xi-Yang Y-B, Wang T-H (2009). Electroacupuncture induced spinal plasticity is linked to multiple gene expressions in dorsal root deafferented rats. *Journal of Molecular Neuroscience*.

[12] Gao Y-Z, Guo S-Y, Yin Q-Z, Hisamitsu T, Jiang X-H (2007). An individual variation study of electroacupuncture analgesia in rats using microarray. *The American Journal of Chinese Medicine*.

[13] Yang L-P, Wang M-C, Liu W-G, Wang M-Q (2007). Effects of warming-needle therapy on gene expression pathways in the patient with knee osteoarthritis of deficiency-cold syndrome. *Zhongguo Zhen Jiu*.

[14] Li M, Zhang Y (2007). Modulation of gene expression in cholesterol-lowering effect of electroacupuncture at Fenglong acupoint (ST40): a cDNA microarray study. *International Journal of Molecular Medicine*.

[15] Chae Y, Park H-J, Hahm D-H, Yi S-H, Lee H (2006). Individual differences of acupuncture analgesia in humans using cDNA microarray. *Journal of Physiological Sciences*.

[16] Wu HG, Liu HR, Zhao C (2005). Study on differentially expressed genes of ulcerative colitis in the rat treated by herbs-partitioned moxibustion. *Zhongguo Zhen Jiu*.

[17] Keun Kim C, Gi SC, Sang DO (2005). Electroacupuncture up-regulates natural killer cell activity: identification of genes altering their expressions in electroacupuncture induced up-regulation of natural killer cell activity. *Journal of Neuroimmunology*.

[18] Guo J-C, Gao H-M, Chen J (2004). Modulation of the gene expression in the protective effects of electroacupuncture against cerebral ischemia: a cDNA microarray study. *Acupuncture and Electro-Therapeutics Research*.

[19] Ko J, Doe SN, Young HL (2002). cDNA microarray analysis of the differential gene expression in the neuropathic pain and electroacupuncture treatment models. *Journal of Biochemistry and Molecular Biology*.

[20] Kim J-H, Kim H-K, Park Y-I (2006). Moxibustion at ST36 alleviates pain in complete Freund’s adjuvant-induced arthritic rats. *The American Journal of Chinese Medicine*.

[21] Qi L, Liu HR, Yi T (2013). Warming moxibustion relieves chronic visceral hyperalgesia in rats: relations to spinal dynorphin and orphanin-FQ system. *Evidence-Based Complementary and Alternative Medicine*.

[22] Yamashita H, Ichiman Y, Tanno Y (2001). Changes in peripheral lymphocyte subpopulations after direct moxibustion. *The American Journal of Chinese Medicine*.

[23] Kung Y-Y, Chen F-P, Hwang S-J (2006). The different immunomodulation of indirect moxibustion on normal subjects and patients with systemic lupus erythematosus. *The American Journal of Chinese Medicine*.

[24] Choi GS, Han JB, Park JH (2004). Effects of moxibustion to zusanli (ST36) on alteration of natural killer cell activity in rats. *The American Journal of Chinese Medicine*.

[25] Han J-B, Oh S-D, Lee K-S (2003). The role of the sympathetic nervous system in moxibustion-induced immunomodulation in rats. *Journal of Neuroimmunology*.

[26] Zhou E-H, Liu H-R, Wu H-G (2009). Down-regulation of protein and mRNA expression of IL-8 and ICAM-1 in colon tissue of ulcerative colitis patients by partition-herb moxibustion. *Digestive Diseases and Sciences*.

[27] Zhou E-H, Wang X-M, Ding G-H (2011). Suspended moxibustion relieves chronic visceral hyperalgesia and decreases hypothalamic corticotropin-releasing hormone levels. *World Journal of Gastroenterology*.

[28] Chen S, Ma S-X (2003). Nitric oxide in the gracile nucleus mediates depressor response to acupuncture (ST36). *Journal of Neurophysiology*.

[29] Stein C, Millan MJ, Herz A (1988). Unilateral inflammation of the hindpaw in rats as a model of prolonged noxious stimulation: alterations in behavior and nociceptive thresholds. *Pharmacology Biochemistry and Behavior*.

[30] Guo Y, Guo H, Zhang L (2005). Genomic analysis of anti-hepatitis B virus (HBV) activity by small interfering RNA and lamivudine in stable HBV-producing cells. *Journal of Virology*.

[31] Patterson TA, Lobenhofer EK, Fulmer-Smentek SB (2006). Performance comparison of one-color and two-color platforms within the MicroArray Quality Control (MAQC) project. *Nature Biotechnology*.

[32] Fang J-Q, Aoki E, Seto A, Yu Y, Kasahara T, Hisamitsu T (1998). Influence of moxibustion on collagen-induced arthritis in mice. *In Vivo*.

[33] Pearson CM, Wood FD (1963). Studies of arthritis and other lesions induced in rats by the injection of mycobacterial adjuvant. VII. Pathologic details of the arthritis and spondylitis. *The American Journal of Pathology*.

[34] Pearson CM, Waksman BH, Sharp JT (1961). Studies of arthritis and other lesions induced in rats by injection of mycobacterial adjuvant. V. Changes affecting the skin and mucous membranes. Comparison of the experimental process with human disease. *The Journal of Experimental Medicine*.

[35] Tang ZL, Song XG, Zang FQ (2003). Study on the action mechanism of moxibustion in anti-inflammation and immunoregulation in rheumatoid arthritis rats. *Acupuncture Research*.

[36] Shi Y, Zhou SL (2003). Effect of zusanli (ST36) acupoint on nerve-endocrine-immune network. *Journal of Jiangxi College of Traditional Chinese Medicine*.

[37] Simon JC (1995). Skin as an immune organ. *Medizinische Monatsschrift für Pharmazeuten*.

[38] Salmon JK, Armstrong CA, Ansel JC (1994). The skin as an immune organ. *Western Journal of Medicine*.

[39] Misery L (1996). The neuro-immuno-cutaneous system (NICS). *Pathologie Biologie*.

[40] Bos JD, Kapsenberg ML (1993). The skin immune system: progress in cutaneous biology. *Immunology Today*.

[41] Misery L (2000). The neuro-immuno-cutaneous system and ultraviolet radiation. *Photodermatology Photoimmunology and Photomedicine*.

[42] Nestle FO, Di Meglio P, Qin J-Z, Nickoloff BJ (2009). Skin immune sentinels in health and disease. *Nature Reviews Immunology*.

[43] Shi Y, Zhou SL (2003). Effect of Zusanli (ST36) Acupoint on nerve-endocrine-immune network. *Journal of Jiangxi College of Traditional Chinese Medicine*.

[44] Li JW, Liu JM, Xiong YY, Xiang SY (2006). Advance in research on nerve-endocrine-immune network of rheumatoid arthritis mediated by acupuncture. *Traditional Chinese Medical Research*.

[45] Jen-Hwey C (2013). How does moxibustion possibly work?. *Evidence-Based Complementary and Alternative Medicine*.

