# Supplementary material for: Gene Expression Profiles at Moxibustioned Site (ST36): A Microarray Analysis
Source: Evid Based Complement Alternat Med. 2013 Sep 22;2013:890579. doi: 10.1155/2013/890579 (PMC3807720; doi:10.1155/2013/890579)
Supplement: Supplementary file 1 — Supplementary Table 1: The DEGs list under physiological condition (CM versus C) Supplementary Table 2: The DEGs list under pathological condition (MM versus M) Supplementary Table 3: Co-expressed genes list between physiological and pathological condition. [file 890579.f1.zip › supplementary data/S1-The DEGs list under physiological condition (CMvsC).doc]

**Supplementary Table 1: The DEGs list under physiological condition (CM vs C)**

| **Oligo_id** | **Name** | **Fold Change** | **Oligo_id** | **Name** | **Fold Change** |
| --- | --- | --- | --- | --- | --- |
| Rn30018562 | Eraf | 18.3971996 | Rn30018393 | Trim8 | 2.510557 |
| Rn30016778 | Ank1 | 15.9722244 | Rn30010795 | Lama3 | 2.485811 |
| Rn30008331 | Mup4 | 15.4240959 | R005067_01 | Rpl3l | 2.483996 |
| Rn30014754 | Ankrd23 | 14.1221342 | Rn30013866 | Lrrc39 | 2.422745 |
| Rn30004769 | Igh-1a | 13.4996318 | Rn30023432 | Krt72 | 2.412589 |
| Rn30000254 | Fn3k | 11.9280769 | Rn30004999 | Mybpc1 | 2.39428 |
| Rn30011179 | Cmya5 | 11.7987646 | Rn30001417 | RGD1559597 | 2.372783 |
| Rn30002404 | Znf692 | 8.63337516 | Rn30018049 | Trpv3 | 2.343382 |
| Rn30007491 | Mylk2 | 8.00232888 | Rn30013897 | Tnnc2 | 2.302247 |
| Rn30011281 | Txlnb | 7.42932151 | Rn30014629 | Lims2 | 2.279703 |
| Rn30004732 | Coch | 7.36808632 | Rn30019573 | Pla2g16 | 2.279435 |
| R003775_01 | Fcnb | 7.33734857 | Rn30025829 | - | 2.271724 |
| R000289_01 | Myf6 | 7.30918408 | R000194_01 | Des | 2.26788 |
| Rn30011801 | C1qa | 7.09975687 | Rn30006570 | Flnc | 2.24064 |
| Rn30000122 | Alas2 | 7.09467317 | R000557_01 | Hspb1 | 2.234037 |
| R002857_01 | Gpr116 | 6.88413864 | Rn30026349 | RGD1308734 | 2.229144 |
| Rn30005739 | LOC298139 | 6.83989103 | Rn30023633 | - | 2.229009 |
| Rn30011659 | Pdlim3 | 6.61419386 | Rn30022911 | Acap2 | 2.210603 |
| Rn30006772 | Gpihbp1 | 6.58211528 | Rn30021099 | Obscnl | 2.19127 |
| R003012_01 | Padi1 | 6.51947189 | Rn30022199 | Phactr1 | 2.178533 |
| Rn30019409 | Lrrc2 | 6.47773068 | R000100_01 | Atp1a2 | 2.162098 |
| Rn30002820 | Mpz | 6.3958254 | Rn30010360 | Tfdp2 | 2.16208 |
| Rn30010067 | LOC361187 | 6.18352024 | Rn30006126 | Chd7 | 2.148528 |
| Rn30013655 | Synpo2 | 6.13209071 | Rn30018688 | Bag3 | 2.129583 |
| Rn30003093 | - | 6.09169902 | Rn30018177 | Actn3 | 2.128644 |
| Rn30010611 | Dusp26 | 5.94867451 | Rn30003550 | Sgca | 2.090168 |
| R000433_01 | Sparcl1 | 5.85179908 | R000118_01 | Rgs5 | 2.087187 |
| Rn30002090 | Kcnj12 | 5.83356699 | Rn30013312 | Coro6 | 2.085912 |
| Rn30007318 | Stac3 | 5.58077556 | Rn30017156 | Ampd1 | 2.076748 |
| Rn30022121 | LOC688915 | 5.51223992 | Rn30021147 | Cnksr1 | 2.066932 |
| Rn30008792 | RGD1308305 | 5.41369601 | Rn30024759 | Armetl1 | 2.0506 |
| Rn30004998 | Mybpc1 | 5.41222349 | Rn30008028 | Nfe2l1 | 2.044142 |
| Rn30013679 | - | 5.27025541 | Rn30000995 | Tesc | 2.039232 |
| Rn30016369 | Atp2b3 | 5.19480081 | R001281_01 | Ldhc | 2.028476 |
| Rn30019656 | RGD1311499 | 5.16060913 | Rn30000308 | Cfb | 2.022483 |
| Rn30011557 | Krtap3*1 | 5.09983243 | R003734_01 | Trpv2 | 2.014405 |
| Rn30018182 | Cyb5r2 | 5.09499261 | Rn30013792 | Tex13b | 0.181003 |
| R004577_01 | Gimap4 | 5.07179219 | Rn30002325 | Serpinb3a | 0.181611 |
| Rn30022801 | LOC501283 | 4.99392388 | Rn30002007 | Hsd17b13 | 0.21654 |
| Rn30013656 | Synpo2 | 4.86878657 | Rn30020479 | Casc4 | 0.217064 |
| Rn30001418 | RGD1565563 | 4.81767572 | Rn30003072 | Cbx8 | 0.21759 |
| R001101_01 | Ppp1r1a | 4.72292025 | Rn30010536 | Sult1c2 | 0.232107 |
| Rn30001571 | Apod | 4.65222009 | Rn30003451 | Rgs13 | 0.240948 |
| Rn30008155 | Obscn | 4.53407069 | Rn30013101 | Rab32 | 0.243729 |
| Rn30011334 | - | 4.52225326 | Rn30002249 | Expi | 0.246569 |
| Rn30019408 | Lrrc2 | 4.51768998 | Rn30015358 | Loxl2 | 0.247664 |
| R001011_01 | Vtn | 4.44536222 | Rn30012061 | Hells | 0.250533 |
| R004305_01 | Cygb | 4.36977638 | Rn30023089 | - | 0.255143 |
| Rn30016367 | - | 4.32555784 | Rn30014041 | Moxd1 | 0.260378 |
| Rn30004305 | Rtn1 | 4.28785736 | Rn30003198 | RGD1564463 | 0.265872 |
| Rn30023415 | Krt73 | 4.09514828 | Rn30019816 | Es22 | 0.271707 |
| Rn30018719 | Tnnt3 | 3.93925386 | R001608_01 | Proc | 0.287454 |
| R001026_01 | Atf3 | 3.93804711 | Rn30004867 | Cdca7l | 0.294382 |
| Rn30002484 | Selp | 3.90808518 | Rn30014500 | Rbp7 | 0.295962 |
| Rn30003890 | - | 3.87791228 | Rn30022351 | - | 0.296009 |
| Rn30002016 | Yipf7 | 3.84775163 | Rn30005947 | Ecop | 0.301439 |
| Rn30004343 | Ptprb | 3.8419649 | Rn30022195 | Skap1 | 0.308338 |
| Rn30012735 | Ankrd2 | 3.81850769 | R001159_01 | Rln1 | 0.311385 |
| R004272_01 | - | 3.80217859 | Rn30026660 | LOC679532 | 0.332887 |
| Rn30025826 | Krtap15*1 | 3.71677089 | Rn30005732 | Samhd1 | 0.337236 |
| Rn30003626 | Kcnc2 | 3.58187242 | Rn30024431 | Ly6d | 0.339877 |
| Rn30015019 | [Pdlim5](http://rgd.mcw.edu/tools/genes/genes_view.cgi?id=621076) | 3.52041056 | Rn30007565 | Myo5a | 0.355005 |
| Rn30007605 | Krt86 | 3.47421709 | Rn30025914 | - | 0.356332 |
| Rn30014544 | Cpxm2 | 3.45722731 | Rn30020974 | - | 0.356528 |
| Rn30003045 | Asb11 | 3.44198869 | Rn30005328 | Slurp1 | 0.356779 |
| Rn30023637 | LOC690097 | 3.40622505 | Rn30012667 | Clca5 | 0.361832 |
| Rn30006462 | RGD1565410 | 3.35945328 | R001665_01 | Ldlr | 0.370746 |
| Rn30023621 | Trdn | 3.29126577 | Rn30017288 | Pmaip1 | 0.372592 |
| Rn30021218 | Emcn | 3.2777796 | Rn30015552 | Igfbp2 | 0.37587 |
| Rn30015312 | Nrap | 3.26189683 | Rn30006534 | Cyp51 | 0.37786 |
| Rn30024809 | Dhrs7c | 3.2378708 | Rn30003184 | Figf | 0.378994 |
| Rn30023636 | Trdn | 3.13348207 | Rn30004020 | Hal | 0.393716 |
| Rn30010544 | Usp13 | 3.12211003 | Rn30018071 | Hacl1 | 0.395123 |
| R000021_01 | Fxyd4 | 3.104129 | Rn30022380 | Sprr2b | 0.397252 |
| Rn30008523 | Nos3 | 3.08797 | R004320_01 | Pdzk1ip1 | 0.398077 |
| Rn30013100 | RGD1307418 | 3.086663 | Rn30012847 | Psat1 | 0.40346 |
| Rn30009615 | Ccno | 3.081477 | Rn30001685 | Klhdc5 | 0.405027 |
| Rn30021616 | Tchh | 3.068377 | Rn30000467 | Pcbd1 | 0.406369 |
| Rn30017451 | Mt3 | 3.062838 | Rn30024669 | RGD1561528 | 0.406977 |
| Rn30004041 | CACNA1B | 3.042304 | Rn30009357 | Tmem9 | 0.410086 |
| Rn30018718 | Tnnt3 | 2.998277 | Rn30016072 | Gja5 | 0.413746 |
| Rn30010838 | Myot | 2.969286 | Rn30014935 | Slc22a1 | 0.415312 |
| R003102_01 | Padi3 | 2.961831 | Rn30024846 | LOC363337 | 0.420527 |
| Rn30012058 | Dusp13 | 2.948859 | Rn30003696 | Pctk2 | 0.421119 |
| Rn30012901 | - | 2.942595 | R000123_01 | Sult1b1 | 0.426658 |
| Rn30002496 | Higd1b | 2.928154 | R004693_01 | Serpinb7 | 0.434263 |
| Rn30015111 | Mbp | 2.918713 | Rn30013184 | anillin | 0.436833 |
| R002935_01 | Kcnj8 | 2.90089 | Rn30012936 | Krt13 | 0.439974 |
| Rn30009378 | Hspb7 | 2.841034 | Rn30008810 | Hsd17b12 | 0.447092 |
| Rn30005972 | Rai2 | 2.794531 | Rn30018070 | Hacl1 | 0.449386 |
| Rn30018310 | Folr2 | 2.786729 | Rn30017595 | RGD1309326 | 0.44952 |
| R003809_01 | Pde4dip | 2.780527 | Rn30024612 | BAT2D1 | 0.452724 |
| Rn30017239 | Pnpla2 | 2.769797 | Rn30011612 | - | 0.453904 |
| Rn30010793 | Sat2 | 2.761595 | Rn30002507 | Hsd17b7 | 0.457081 |
| Rn30019462 | Tmod4 | 2.749823 | Rn30008956 | Krt8 | 0.457967 |
| Rn30010458 | Ldb3 | 2.743762 | Rn30012206 | Cyp2c23 | 0.458605 |
| Rn30006833 | Angptl4 | 2.709884 | Rn30003747 | Dtl | 0.462375 |
| Rn30007729 | Nfasc | 2.67198 | Rn30009700 | Srpr | 0.464802 |
| Rn30024874 | RGD1563334 | 2.664357 | Rn30007929 | Slc37a3 | 0.465534 |
| Rn30016744 | Slc28a2 | 2.635653 | Rn30005229 | - | 0.469092 |
| Rn30015001 | - | 2.608263 | Rn30008710 | Pgp | 0.471723 |
| R002436_01 | Pdlim3 | 2.596843 | Rn30010293 | Wdhd1 | 0.472643 |
| R001136_01 | Gp5 | 2.588646 | Rn30011015 | Ndufb5 | 0.47338 |
| Rn30022041 | Hnt | 2.588417 | Rn30001190 | Zfp68 | 0.473432 |
| R003366_01 | Slc9a3r2 | 2.569533 | Rn30017247 | Ugt1a3 | 0.474866 |
| Rn30017585 | Hmgcs2 | 2.54341 | Rn30013264 | Krt16 | 0.479544 |
| R004231_01 | Bcl2l10 | 2.538298 | R000908_01 | Slc25a1 | 0.479581 |
| Rn30012686 | C1qtnf9 | 2.519334 | R004905_01 | Cog8 | 0.497984 |
| Rn30014959 | Colec12 | 2.515977 |  |  |  |

Note:- unknown.
