# Supplementary material for: Gene Expression Profiles at Moxibustioned Site (ST36): A Microarray Analysis
Source: Evid Based Complement Alternat Med. 2013 Sep 22;2013:890579. doi: 10.1155/2013/890579 (PMC3807720; doi:10.1155/2013/890579)
Supplement: Supplementary file 1 — Supplementary Table 1: The DEGs list under physiological condition (CM versus C) Supplementary Table 2: The DEGs list under pathological condition (MM versus M) Supplementary Table 3: Co-expressed genes list between physiological and pathological condition. [file 890579.f1.zip › supplementary data/S2-The DEGs list under pathological condition (MMvsM).doc]

**Supplementary Table 2:The DEGs list under pathological condition (MM vs M)**

| **Oligo_id** | **Name** | **Fold Change** | **Oligo_id** | **Name** | **Fold Change** |
| --- | --- | --- | --- | --- | --- |
| Rn30018562 | Eraf | 41.083244 | Rn30004066 | Fam36a | 2.057539 |
| Rn30000745 | Hspa1b | 27.571363 | Rn30010875 | RGD1560323 | 2.054533 |
| Rn30018964 | Mcpt8 | 22.952380 | Rn30004333 | - | 2.041587 |
| Rn30008354 | Krt75 | 21.734422 | Rn30014802 | MGC94207 | 2.038315 |
| Rn30002705 | - | 17.032027 | Rn30000366 | Pfdn6 | 2.037241 |
| Rn30005152 | Dapl1 | 16.453815 | Rn30001820 | - | 2.004426 |
| Rn30002678 | - | 14.932310 | Rn30011801 | C1qa | 0.018447 |
| Rn30016143 | Obp3 | 14.916883 | Rn30008196 | Mmp3 | 0.020423 |
| R002437_01 | Tm4sf4 | 14.202362 | R003960_01 | Csf3 | 0.021640 |
| Rn30008966 | Cdkn3 | 13.251539 | Rn30008190 | Mmp3 | 0.029146 |
| Rn30022203 | RGD1564658 | 12.376319 | Rn30008192 | Mmp3 | 0.052190 |
| Rn30016778 | Ank1 | 11.486481 | R002729_01 | Cyr61 | 0.073092 |
| Rn30018284 | RGD1565787 | 11.370570 | Rn30012770 | Slpi | 0.080789 |
| Rn30025543 | LOC500736 | 11.126715 | R002336_01 | - | 0.082856 |
| Rn30021953 | - | 11.019629 | Rn30002157 | Prg4 | 0.087210 |
| Rn30011324 | - | 10.671160 | Rn30026013 | Lilrb4 | 0.095889 |
| Rn30005130 | Spic | 10.283387 | Rn30017416 | - | 0.099693 |
| Rn30013296 | - | 10.004695 | Rn30002049 | - | 0.113275 |
| Rn30015151 | Ns5atp9 | 9.343661 | R001406_01 | Ccl3 | 0.116901 |
| Rn30003045 | Asb11 | 9.035264 | Rn30023219 | Cxcl13 | 0.118303 |
| Rn30004711 | Srl | 8.773841 | Rn30022370 | - | 0.119984 |
| Rn30014222 | Ces3 | 8.597923 | R002312_01 | Pla2g2a | 0.123126 |
| Rn30004655 | - | 8.488780 | Rn30026019 | Lilrb4 | 0.123679 |
| Rn30018835 | Tacc2 | 8.478818 | Rn30004446 | - | 0.125408 |
| Rn30006073 | Smyd1 | 8.389335 | R004548_01 | Ppp1r14c | 0.140896 |
| Rn30014233 | Cdca3 | 8.346326 | Rn30017247 | Ugt1a1 | 0.146449 |
| Rn30017593 | - | 8.303850 | Rn30022462 | - | 0.146564 |
| Rn30026350 | - | 8.133881 | Rn30015878 | Igsf6 | 0.152969 |
| Rn30022958 | Ckap2 | 8.055811 | Rn30008193 | Mmp10 | 0.156649 |
| Rn30006313 | RGD1304693 | 7.887529 | Rn30019531 | Ctss | 0.166889 |
| Rn30016369 | Atp2b3 | 7.652867 | Rn30022690 | Fcgr3a | 0.168175 |
| Rn30021553 | Ear11 | 7.595964 | R002882_01 | Timp1 | 0.168222 |
| Rn30011179 | - | 7.549108 | Rn30007566 | Myo5a | 0.169139 |
| Rn30009494 | Ear11 | 7.469758 | Rn30002801 | LOC498277 | 0.169486 |
| Rn30002673 | - | 7.418829 | Rn30023425 | - | 0.172619 |
| Rn30005086 | Aurkb | 7.393346 | Rn30024065 | - | 0.173695 |
| Rn30003246 | Igj | 7.328226 | Rn30022401 | - | 0.176577 |
| Rn30020892 | LOC364773 | 7.163163 | Rn30020062 | Clec4a1 | 0.176636 |
| Rn30020334 | RGD1310251 | 7.161664 | Rn30002797 | Fcgr2b | 0.176964 |
| R000142_01 | Cdh1 | 7.051433 | Rn30026040 | - | 0.180920 |
| Rn30016930 | - | 6.964460 | Rn30008293 | - | 0.182392 |
| R002501_01 | - | 6.955927 | Rn30014857 | Cotl1 | 0.185676 |
| Rn30000254 | Fn3k | 6.851828 | Rn30009651 | Dnajb14 | 0.186765 |
| R000038_01 | Pttg1 | 6.785596 | Rn30020063 | Clec4a1 | 0.187756 |
| Rn30007675 | RGD1559671 | 6.743277 | Rn30020273 | - | 0.192524 |
| Rn30006807 | Smtnl1 | 6.707915 | R003618_01 | Slc2a3 | 0.193773 |
| R002677_01 | Nkg7 | 6.615996 | Rn30013130 | Stk11 | 0.195048 |
| R004692_01 | Lhx2 | 6.597168 | Rn30005980 | Nedd4 | 0.195602 |
| Rn30017136 | Ccnb1 | 6.334441 | Rn30012017 | Prc1 | 0.197535 |
| R004465_01 | Pcdh21 | 6.322125 | Rn30026876 | Slc28a2 | 0.197758 |
| Rn30021366 | - | 6.304579 | Rn30023067 | - | 0.202003 |
| Rn30020465 | Ctdspl2 | 6.287201 | Rn30017269 | - | 0.202503 |
| Rn30018960 | Efna4 | 6.260781 | Rn30011163 | - | 0.202811 |
| Rn30021747 | - | 6.247623 | Rn30001094 | - | 0.203318 |
| Rn30000067 | Rnase17 | 6.225851 | Rn30012434 | - | 0.212644 |
| Rn30026814 | Bex4 | 6.224025 | Rn30002325 | Serpinb3a | 0.213768 |
| Rn30006268 | Ctse | 6.163654 | Rn30001575 | Eif4g1 | 0.217571 |
| Rn30014030 | Pbk | 6.079240 | Rn30014962 | Ppp1r14c | 0.222565 |
| Rn30002090 | Kcnj12 | 5.868604 | Rn30024709 | - | 0.224277 |
| Rn30009390 | Chmp4c | 5.778545 | Rn30011612 | - | 0.227387 |
| Rn30000844 | - | 5.760419 | Rn30016785 | - | 0.227431 |
| R004229_01 | Ppbp | 5.743860 | Rn30005732 | Samhd1 | 0.233371 |
| R004509_01 | Adipoq | 5.721382 | Rn30006757 | Serping1 | 0.233464 |
| Rn30011659 | Pdlim3 | 5.720480 | Rn30016806 | - | 0.235228 |
| R005310_01 | Hsph1 | 5.706119 | Rn30024525 | Hk3 | 0.241092 |
| Rn30004899 | - | 5.702579 | Rn30004547 | Zfp36l2 | 0.243881 |
| Rn30016016 | - | 5.692046 | Rn30008365 | - | 0.244786 |
| Rn30010964 | Rasa2 | 5.657670 | Rn30013792 | Tex13b | 0.244929 |
| Rn30020948 | - | 5.623890 | Rn30011731 | - | 0.245477 |
| Rn30011587 | Tlcd1 | 5.566018 | R001889_01 | Itgb7 | 0.245665 |
| Rn30016246 | Dennd2d | 5.471729 | Rn30011035 | C1s | 0.249626 |
| Rn30025297 | Slc5a3 | 5.417747 | R003495_01 | Cd14 | 0.250696 |
| Rn30019056 | Gzmb | 5.384253 | Rn30006408 | - | 0.257104 |
| Rn30003464 | Col1a1 | 5.377252 | Rn30004453 | Phf15 | 0.258588 |
| Rn30004900 | RGD1561776 | 5.356750 | R000009_01 | Enpp3 | 0.259257 |
| Rn30023050 | - | 5.349726 | Rn30006438 | Myo1g | 0.260811 |
| Rn30003462 | Col1a1 | 5.330135 | Rn30017105 | Ankrd1 | 0.261574 |
| Rn30023060 | - | 5.329166 | Rn30009012 | - | 0.263812 |
| Rn30024809 | Dhrs7c | 5.310138 | Rn30005979 | Nedd4 | 0.263944 |
| Rn30002820 | Mpz | 5.302353 | Rn30019958 | - | 0.265354 |
| Rn30006081 | - | 5.295778 | Rn30007801 | Batf | 0.266631 |
| Rn30024948 | - | 5.179125 | R003916_01 | - | 0.266748 |
| Rn30002295 | Tnn | 5.174581 | Rn30007882 | - | 0.267612 |
| Rn30015724 | Pacrg | 5.167651 | Rn30011246 | Itgb7 | 0.270174 |
| Rn30014003 | RGD1310784 | 5.123489 | Rn30013737 | Slc11a1 | 0.273338 |
| Rn30013876 | Ube2c | 5.080434 | Rn30002798 | Fcgr3 | 0.274118 |
| Rn30011451 | - | 5.048328 | Rn30019473 | Tnfaip8l2 | 0.274989 |
| Rn30018851 | Wnk4 | 5.043229 | Rn30017219 | Mgl1 | 0.275313 |
| Rn30006521 | Hspcal3 | 5.035602 | Rn30004279 | Myf5 | 0.278035 |
| Rn30001969 | RGD1310958 | 4.892431 | Rn30022430 | - | 0.278644 |
| R004336_01 | Vegfc | 4.858425 | Rn30022466 | - | 0.279795 |
| Rn30005567 | Klhl31 | 4.852520 | Rn30019773 | Slfn8 | 0.281728 |
| Rn30013155 | - | 4.831596 | Rn30020823 | - | 0.283517 |
| Rn30017686 | Rhod | 4.710167 | Rn30006415 | Lgmn | 0.286307 |
| R004106_01 | Nr4a3 | 4.680434 | R001657_01 | Il4ra | 0.292119 |
| Rn30019936 | - | 4.668682 | Rn30025515 | Aph1b | 0.294235 |
| Rn30014807 | Micalcl | 4.643221 | Rn30004105 | Galntl1 | 0.298191 |
| Rn30023621 | Trdn | 4.639911 | Rn30016804 | Errfi1 | 0.302994 |
| Rn30022390 | Fbxo5 | 4.576998 | R001297_01 | Apobec1 | 0.304338 |
| R000042_01 | Stmn1 | 4.555419 | Rn30014070 | Aox1 | 0.304623 |
| Rn30020026 | Dcakd | 4.509214 | R002937_01 | Serpine1 | 0.311229 |
| Rn30024343 | Grem1 | 4.503192 | Rn30018768 | - | 0.312202 |
| Rn30017451 | Mt3 | 4.492259 | Rn30008505 | Hck | 0.313304 |
| Rn30008641 | - | 4.474888 | Rn30026753 | - | 0.315472 |
| Rn30021004 | - | 4.449336 | Rn30023637 | - | 0.315689 |
| Rn30019475 | Scn1b | 4.407642 | Rn30008846 | Tnnt2 | 0.317458 |
| Rn30018668 | Kif22 | 4.398701 | Rn30014156 | - | 0.319465 |
| Rn30006390 | Atp1b4 | 4.203583 | R002652_01 | Ifngr1 | 0.321670 |
| R001375_01 | - | 4.120092 | Rn30009206 | Clecsf6 | 0.321890 |
| Rn30022142 | Fndc5 | 4.094028 | Rn30022563 | Plaur | 0.326060 |
| Rn30007828 | Mal2 | 4.062913 | R002843_01 | Gda | 0.326300 |
| Rn30012901 | - | 3.994770 | Rn30000608 | - | 0.328782 |
| Rn30016881 | Nt5dc2 | 3.934200 | Rn30020329 | - | 0.331149 |
| Rn30023140 | - | 3.838821 | R000080_01 | Cst3 | 0.333240 |
| Rn30002155 | Mfap4 | 3.775512 | Rn30000186 | - | 0.337909 |
| Rn30018211 | Dyrk4 | 3.760977 | Rn30012836 | Il6st | 0.341391 |
| Rn30004691 | Tcfap2c | 3.725994 | Rn30009586 | Dync1li1 | 0.341404 |
| Rn30002016 | Yipf7 | 3.716659 | R004525_01 | Psmb8 | 0.343032 |
| Rn30018961 | - | 3.665483 | Rn30006689 | Per1 | 0.343255 |
| Rn30008218 | Asb2 | 3.624158 | Rn30013701 | - | 0.344315 |
| Rn30003648 | Trib1 | 3.614713 | Rn30001184 | Fam20c | 0.344458 |
| Rn30023927 | - | 3.607788 | R002180_01 | Eif4ebp1 | 0.345158 |
| Rn30015077 | RGD1565202 | 3.586203 | R004337_01 | Litaf | 0.346097 |
| R002663_01 | Hspb3 | 3.582775 | R004187_01 | Fgl2 | 0.346351 |
| Rn30020690 | RGD1565395 | 3.537159 | Rn30000609 | - | 0.347283 |
| Rn30015282 | Idi1 | 3.533668 | Rn30016744 | Slc28a2 | 0.349172 |
| R000447_01 | Wfdc1 | 3.528646 | R000055_01 | Pdpn | 0.350188 |
| Rn30007732 | Dbf4 | 3.481672 | R004620_01 | Cryab | 0.350700 |
| Rn30017437 | Tnnc1 | 3.462212 | Rn30000541 | Ptprc | 0.351050 |
| R005385_01 | Drg1 | 3.455561 | Rn30013132 | Fn1 | 0.359370 |
| Rn30012085 | - | 3.446518 | Rn30007859 | Igfbp3 | 0.361284 |
| Rn30015226 | - | 3.425474 | R002658_01 | Fcna | 0.362465 |
| Rn30000013 | Tcf15 | 3.391982 | Rn30009900 | Aig1 | 0.364591 |
| R003568_01 | Jun | 3.387341 | Rn30012429 | Rassf4 | 0.365033 |
| Rn30021710 | RGD1561694 | 3.375652 | Rn30020829 | Fkbp5 | 0.367902 |
| R000524_01 | Hpd | 3.373605 | Rn30000167 | Ccl7 | 0.368221 |
| Rn30009002 | Ttk | 3.363693 | Rn30000963 | Zdhhc4 | 0.369900 |
| Rn30009399 | - | 3.339260 | Rn30015171 | Serpinb1a | 0.370152 |
| Rn30000432 | - | 3.307280 | Rn30017075 | Ctsl | 0.371382 |
| Rn30014500 | Rbp7 | 3.306085 | Rn30014231 | Pja2 | 0.373157 |
| Rn30004743 | - | 3.248698 | Rn30015177 | Ninj1 | 0.376058 |
| Rn30011831 | - | 3.212814 | Rn30016926 | - | 0.377316 |
| Rn30010060 | Ermp1 | 3.191089 | Rn30013573 | - | 0.378261 |
| Rn30008280 | Prkar2b | 3.187954 | Rn30011077 | B3galnt1 | 0.378857 |
| Rn30011533 | - | 3.173498 | Rn30000668 | - | 0.384845 |
| Rn30021092 | Pphln1 | 3.172590 | Rn30010874 | Sgk1 | 0.385900 |
| Rn30009941 | Esm1 | 3.145577 | Rn30016528 | Serpinb6a | 0.387524 |
| R002900_01 | Tnmd | 3.132247 | Rn30016793 | Ampd3 | 0.389024 |
| Rn30015424 | Cmtm5 | 3.131047 | Rn30024749 | Ppap2a | 0.389570 |
| Rn30011141 | Ltbp2 | 3.117497 | R000368_01 | Vim | 0.391799 |
| R000222_01 | Rgs2 | 3.111061 | Rn30016235 | - | 0.392560 |
| Rn30001287 | Cux1 | 3.103367 | Rn30000362 | RT1-A2 | 0.395508 |
| Rn30018684 | Usmg5 | 3.088809 | R000068_01 | Fmo1 | 0.395835 |
| Rn30010579 | - | 3.083949 | Rn30005619 | - | 0.396010 |
| Rn30016179 | Srd5a1 | 3.075238 | Rn30002249 | Expi | 0.396217 |
| Rn30002459 | Areg | 3.066883 | Rn30002059 | Naaa | 0.396580 |
| Rn30018467 | Tex101 | 3.047022 | Rn30018080 | Pgpep1 | 0.398860 |
| Rn30018070 | Hacl1 | 3.025972 | Rn30012174 | Sdccag10 | 0.399389 |
| Rn30019310 | Capn1 | 3.018641 | R000806_01 | Emp3 | 0.399617 |
| Rn30009609 | - | 3.010926 | R003915_01 | Hspb8 | 0.400687 |
| Rn30014572 | Bub1 | 2.981897 | R001319_01 | Mvp | 0.401162 |
| R001836_01 | Cpa3 | 2.973647 | R001476_01 | Adm | 0.401639 |
| Rn30022620 | RGD1565350 | 2.966221 | Rn30005642 | Ptges | 0.402358 |
| R003045_01 | Omd | 2.944932 | R004277_01 | Tmem49 | 0.406021 |
| R000968_01 | S100b | 2.928927 | Rn30018564 | Reep5 | 0.406500 |
| Rn30008506 | RGD1311874 | 2.922055 | Rn30019436 | Fxyd5 | 0.406858 |
| R005101_01 | Stard10 | 2.915885 | Rn30010858 | C1r | 0.407150 |
| R003150_01 | Sqle | 2.914396 | R001662_01 | Ptprc | 0.411062 |
| Rn30017622 | RGD1308113 | 2.909244 | R002625_01 | Jak2 | 0.414560 |
| Rn30026696 | Pak1 | 2.908412 | Rn30019113 | Cdc25a | 0.414820 |
| Rn30006896 | C1qtnf5 | 2.903335 | Rn30006391 | Adfp | 0.416464 |
| Rn30018049 | Trpv3 | 2.873781 | R002604_01 | Alox5ap | 0.417893 |
| Rn30003785 | Pole2 | 2.872745 | Rn30003498 | - | 0.418772 |
| Rn30024466 | Parva | 2.856097 | Rn30020794 | Plp2 | 0.419623 |
| Rn30000995 | Tesc | 2.841624 | Rn30002177 | RGD1359713 | 0.420936 |
| Rn30016524 | Golga7 | 2.832906 | Rn30024944 | - | 0.421926 |
| Rn30013879 | - | 2.829917 | Rn30013301 | Smc5 | 0.422578 |
| Rn30008047 | Ttc5 | 2.825991 | R000212_01 | Tspo | 0.422600 |
| R002962_01 | Amd1 | 2.822354 | Rn30025127 | - | 0.423429 |
| Rn30014595 | - | 2.818882 | Rn30003742 | - | 0.423892 |
| Rn30018347 | Rhot2 | 2.816808 | Rn30004208 | - | 0.425732 |
| Rn30001810 | - | 2.796143 | Rn30018690 | Lsp1 | 0.426702 |
| Rn30014276 | - | 2.784631 | Rn30023787 | Wdr41 | 0.426859 |
| Rn30021967 | - | 2.777963 | Rn30003090 | Ifi204 | 0.426872 |
| Rn30024620 | RGD1304876 | 2.759393 | Rn30024110 | Cfp | 0.428124 |
| Rn30000244 | - | 2.751578 | Rn30017240 | Pnpla2 | 0.429275 |
| R004431_01 | Selenbp1 | 2.739851 | Rn30000361 | - | 0.429775 |
| Rn30007326 | - | 2.727273 | Rn30003243 | - | 0.430148 |
| Rn30021040 | - | 2.709343 | R002756_01 | Fst | 0.434164 |
| Rn30017232 | Rad9 | 2.705835 | R002856_01 | Map4k3 | 0.435397 |
| Rn30006911 | Zmynd17 | 2.704061 | Rn30024020 | - | 0.436177 |
| Rn30007758 | Pdlim2 | 2.687595 | R003898_01 | Bid | 0.437166 |
| Rn30006157 | - | 2.678904 | Rn30000743 | Fhl1 | 0.437220 |
| Rn30013029 | - | 2.674300 | Rn30006692 | Nfkbia | 0.437327 |
| R003656_01 | Gpd1 | 2.664541 | Rn30001856 | LOC289482 | 0.437490 |
| Rn30012424 | Mat2a | 2.643980 | Rn30022686 | - | 0.438849 |
| Rn30005463 | Lynx1 | 2.628148 | Rn30006244 | Arhgap9 | 0.441880 |
| Rn30014755 | Ankrd23 | 2.605204 | Rn30015393 | Phyhd1 | 0.442472 |
| R000432_01 | Lum | 2.582738 | Rn30015029 | Tyr | 0.443632 |
| R004038_01 | - | 2.564791 | Rn30012141 | Hmha1 | 0.444503 |
| Rn30019238 | Tdrkh | 2.547205 | Rn30012210 | Mgst2 | 0.444838 |
| Rn30010472 | Slc37a4 | 2.539644 | R000942_01 | Fez2 | 0.447250 |
| R002978_01 | Hspa4 | 2.530917 | Rn30009825 | Wisp2 | 0.447318 |
| Rn30018297 | Matr3 | 2.468580 | Rn30010542 | RGD1561205 | 0.448085 |
| R005497_01 | Mlf1 | 2.422589 | Rn30014232 | Pja2 | 0.449046 |
| Rn30001603 | Tfrc | 2.414134 | Rn30020811 | - | 0.451590 |
| R003751_01 | Scg2 | 2.408769 | Rn30004700 | - | 0.452181 |
| Rn30008176 | Irak1bp1 | 2.405833 | R002584_01 | Nr2f2 | 0.452891 |
| Rn30026657 | - | 2.398906 | Rn30017768 | Tspan4 | 0.453948 |
| R005080_01 | Tm9sf3 | 2.392445 | R003575_01 | Batf3 | 0.453988 |
| Rn30019324 | Pthr1 | 2.387292 | Rn30015949 | Fbxw4 | 0.454251 |
| Rn30021903 | - | 2.386393 | Rn30008719 | Rpo1-4 | 0.454915 |
| Rn30009296 | Pdcd10 | 2.378538 | R000450_01 | Slc2a1 | 0.455179 |
| R003400_01 | Mb | 2.364087 | Rn30006831 | - | 0.456476 |
| Rn30000189 | - | 2.360489 | Rn30005065 | RGD1564065 | 0.458225 |
| Rn30001405 | Atp5j | 2.341693 | Rn30000360 | RT1-A3 | 0.460989 |
| Rn30018460 | Slc7a6os | 2.324999 | Rn30009697 | Mrpl45 | 0.461327 |
| Rn30008261 | Sec62 | 2.304420 | Rn30016002 | Apob48r | 0.463604 |
| Rn30010601 | Pick1 | 2.300075 | Rn30003601 | - | 0.465082 |
| Rn30008039 | Orc1l | 2.298594 | Rn30009029 | Il18 | 0.467233 |
| Rn30010276 | Arsb | 2.282641 | R004296_01 | Carhsp1 | 0.470142 |
| Rn30014397 | Parva | 2.278451 | Rn30008323 | Mad2l2 | 0.470532 |
| Rn30010270 | - | 2.275979 | Rn30006528 | - | 0.470765 |
| Rn30010677 | - | 2.266042 | Rn30011252 | - | 0.474175 |
| Rn30018587 | - | 2.263712 | Rn30013053 | Fam119a | 0.475317 |
| R002892_01 | Car2 | 2.249938 | Rn30016375 | LOC39692 | 0.477302 |
| Rn30019356 | Tm7sf2 | 2.230323 | Rn30000298 | Gopc | 0.477984 |
| Rn30018311 | Sgta | 2.221152 | R000678_01 | Grn | 0.479389 |
| Rn30019269 | Dpf2 | 2.216393 | Rn30015017 | Rnh1 | 0.479422 |
| Rn30021571 | Cenpm | 2.215065 | Rn30025372 | - | 0.480885 |
| Rn30006523 | Dut-ps | 2.208066 | Rn30000352 | Hla-dma | 0.481522 |
| Rn30015559 | - | 2.201706 | R003693_01 | Slco2b1 | 0.481547 |
| Rn30009659 | - | 2.195713 | Rn30012728 | Arhgap17 | 0.482323 |
| Rn30000773 | Uspl1 | 2.174628 | R000846_01 | Ripk3 | 0.482466 |
| Rn30004914 | - | 2.171575 | Rn30015819 | Tcirg1 | 0.482773 |
| Rn30013007 | Rbm15b | 2.137839 | Rn30002405 | Lamc1 | 0.483298 |
| Rn30011001 | Abhd14a | 2.137585 | Rn30008905 | Praf2 | 0.483707 |
| Rn30016600 | Mrpl48 | 2.127500 | Rn30011774 | - | 0.486360 |
| Rn30009109 | Cib2 | 2.122372 | Rn30018582 | Ahnak | 0.486533 |
| Rn30004521 | Ube2t | 2.119026 | Rn30001421 | - | 0.486746 |
| Rn30010641 | Sub1 | 2.118062 | Rn30003602 | Glipr1 | 0.487234 |
| Rn30015911 | RGD1359529 | 2.117040 | Rn30007540 | - | 0.491614 |
| Rn30001332 | Eln | 2.112027 | R000612_01 | - | 0.492266 |
| Rn30007194 | Cgref1 | 2.092496 | Rn30024910 | - | 0.492421 |
| Rn30021651 | - | 2.081677 | R002276_01 | Lcn2 | 0.494408 |
| R002109_01 | Rgs4 | 2.072322 | Rn30010179 | Laptm5 | 0.495186 |
| Rn30011282 | RGD1305288 | 2.065939 | Rn30010425 | Uhrf2 | 0.495417 |
| Rn30017130 | Plekhb1 | 2.063137 | R004088_01 | Eef2 | 0.498952 |
| R003734_01 | Trpv2 | 2.061259 | R000911_01 | Abcg1 | 0.499568 |

Note:- unknown.
