# Supplementary material for: Gene Expression Profiles at Moxibustioned Site (ST36): A Microarray Analysis
Source: Evid Based Complement Alternat Med. 2013 Sep 22;2013:890579. doi: 10.1155/2013/890579 (PMC3807720; doi:10.1155/2013/890579)
Supplement: Supplementary file 1 — Supplementary Table 1: The DEGs list under physiological condition (CM versus C) Supplementary Table 2: The DEGs list under pathological condition (MM versus M) Supplementary Table 3: Co-expressed genes list between physiological and pathological condition. [file 890579.f1.zip › supplementary data/S3-Co-expressed genes list between physiological and pathological condition .doc]

**Supplementary Table 3: Co-expressed genes list between physiological and pathological condition**

| **DEG_Overlap** | **Oligo_id** | **Name** | **Fold Change（CM/C)** | **Fold Change(MM/M)** |
| --- | --- | --- | --- | --- |
| **Co-upregulated** | Rn30018562 | Eraf | 18.397200 | 41.083244 |
|  | Rn30016778 | Ank1 | 15.972224 | 11.486481 |
|  | Rn30003045 | Asb11 | 3.441989 | 9.035264 |
|  | Rn30016369 | Atp2b3 | 5.194801 | 7.652867 |
|  | Rn30011179 | - | 11.798765 | 7.549108 |
|  | Rn30000254 | Fn3k | 11.928077 | 6.851828 |
|  | Rn30002090 | Kcnj12 | 5.833567 | 5.868604 |
|  | Rn30011659 | Pdlim3 | 6.614194 | 5.720480 |
|  | Rn30024809 | Dhrs7c | 3.237871 | 5.310138 |
|  | Rn30002820 | Mpz | 6.395825 | 5.302353 |
|  | Rn30023621 | Trdn | 3.291266 | 4.639911 |
|  | Rn30017451 | Mt3 | 3.062838 | 4.492259 |
|  | Rn30002016 | Yipf7 | 3.847752 | 3.716659 |
|  | Rn30018049 | Trpv3 | 2.343382 | 2.873781 |
|  | Rn30000995 | Tesc | 2.039232 | 2.841624 |
|  | R003734_01 | Trpv2 | 2.014405 | 2.061259 |
| **Co-downregulated** | Rn30002249 | Expi | 0.246569 | 0.396217 |
|  | Rn30013792 | Tex13b | 0.181003 | 0.244929 |
|  | Rn30005732 | Samhd1 | 0.337236 | 0.233371 |
|  | Rn30011612 | - | 0.453904 | 0.227387 |
|  | Rn30002325 | Serpinb3a | 0.181611 | 0.213768 |
|  | Rn30017247 | Ugt1a1 | 0.474866 | 0.146449 |

Note:- unknown.
